# Technical aspects of endoscopic internal drainage procedure, secured by endoscopic suture fixation: Experimental study

**DOI:** 10.1055/a-2777-9441

**Published:** 2026-01-14

**Authors:** Joel Troya, Karl-Hermann Fuchs, Alexander Hann, Alexander Meining

**Affiliations:** 1 368109 Gastroenterology, Universitätsklinikum Würzburg, Medizinische Klinik und Poliklinik II, Würzburg, Germany; 2 27207 Interventional and Experimental Endoscopy (InExEn), Department of Internal Medicine 2, Universitätsklinikum Würzburg, Würzburg, Germany

**Keywords:** Endoscopy Upper GI Tract, Endoscopic resection (ESD, EMRc, ...), Dilation, injection, stenting, GI surgery

## Abstract

Techniques of interventional endoscopy such as implantation of stents, leak closure by clips, or endoscopic suturing can help in reducing risk of an unfavorable outcome for patients with fistulas in the gastrointestinal tract. One method is endoscopic internal drainage (EID), which has been reported to have remarkable success. Because dislocation can reduce success, endoscopic suture techniques have been applied; however, devices could be cumbersome and/or expensive. The purpose of this experimental study was to evaluation the new endoscopic suturing needle-holder SutuArt for fixation of internal drains at a gastric fistula site. This suturing system is a through-the-scope needle-holder, which can be rotated within the working channel 360 degrees and maneuvered with the endoscope tip in many positions. The experiment was performed using an explanted porcine stomach with attached esophagus. Three consecutive running stitches were performed to provide sufficient fixation of the drain at an experimental “fistula” site. Afterward, the force was measured to dislocate the fixed drain. The results of 12 measurements (median duration 23 minutes; range: 19–44) at 6.7 Newton were compared with the reference value of 12 Newton (full-thickness open-stitch), thus withstanding a substantial pulling force. In conclusion, this study demonstrates the conceptual possibility of using an endoscopic needle holder for suture-fixation of a drain. Further clinical investigations are required to establish a full feasibility test of the concept.

## Introduction

Interventional endoscopic techniques such as stent implantation, leak closure by clips or endoscopic suturing, and endoscopic vacuum therapy have helped to reduce unfavorable outcomes in patients with leaks and fistulas 1 2 3 . If initial leak closure fails, effective wound drainage from the fistula or abscess cavity is important. At present, several suture systems for closure of fistulas are available, but failure may occur 1 4 5 6 7 .

Placement of a sponge at the leak and/or in a cavity associated with vacuum therapy can create sufficient drainage of fluid, providing a chance to develop granulation and eventually healing 1 2 3 . However, the need for persistent drainage usually through the nasopharynx is an annoying option for patients. Endoscopic stent therapy is an attractive alternative; however, insufficient drainage and stent migration often limit its success 1 3 .

Successful modifications of drainage techniques have been reported by Loske in assembling a personalized drainage system, associated with external suction 2 . Another successful therapy has been reported in esophageal leaks: placing endoscopic internal drainage (EID) without suction 8 . This facilitates treatment of patients, because their quality of life may be better without permanent nasogastric tubes.

The idea emerged that the latter two techniques could possibly be combined, by placing a personalized internal drain to avoid the annoying nasogastric tube. However, EID migration may occur in 10% to 15% of patients 1 3 8 . Again, several suture systems and clip applications are available to secure drains in their position 4 5 6 7 9 .

Therefore, we planned to study the technical principle of using a new endoscopic suturing device (SutuArt, Olympus-Medical-Systems-Corp OMSC) to fix the position of a Foley-combined drain at a fistula site by simply suturing the drain to the gut wall at this position and evaluating the durability of this fixation.

## Patients and methods

The main focus of this experimental study was to explore whether an endoscopically placed drain in a simulated fistula can be fixed at the gastric wall using the new endoscopic needle holder reliably enough to withstand even a mechanically applied pulling force to keep the drain in position and prevent dislocation and migration.

**Fig. 1:**
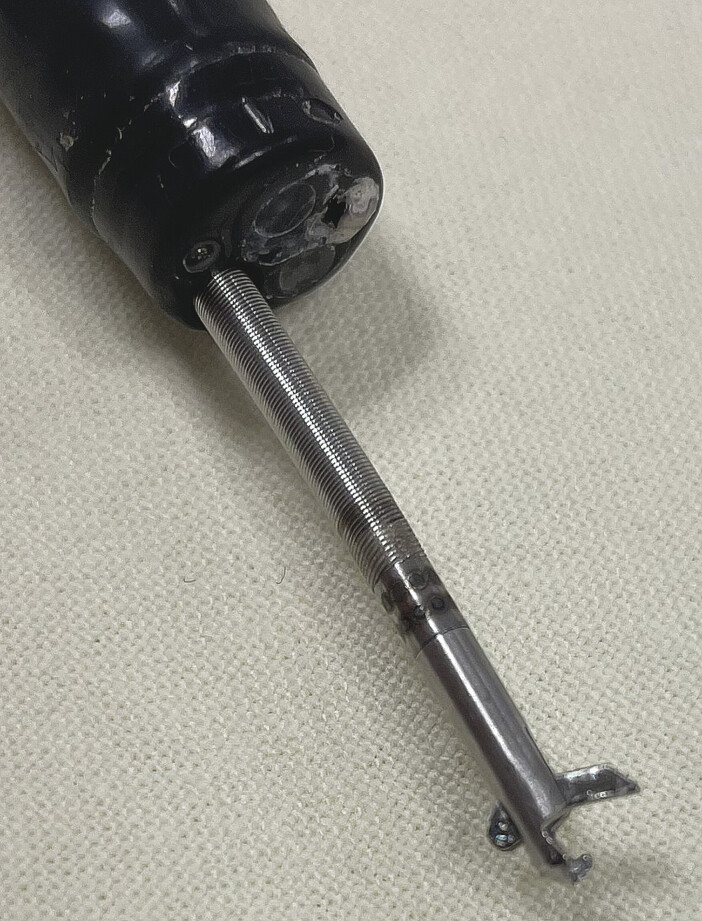
Endoscopic needle-holder applicable through-the-scope SutuArt by Olympus Corporation Tokyo, Japan.

The new suturing system is a through-the-scope needle holder, which can be rotated within the working channel 360 degrees as well as maneuvered in its position together with the endoscope tip in many positions ( Fig. 1 ). A regular gastroscope GIF-H180(OMSC) was used. For suturing, a V-LOC suture material (Covidien/Medtronic, Dublin, Ireland) was applied. This is a monofilament absorbable thread armed with unidirectional barbs, connected to a taper-pointed tip with a semicircular needle, which has been applied for running suture tissue adaptation.

**Fig. 2:**
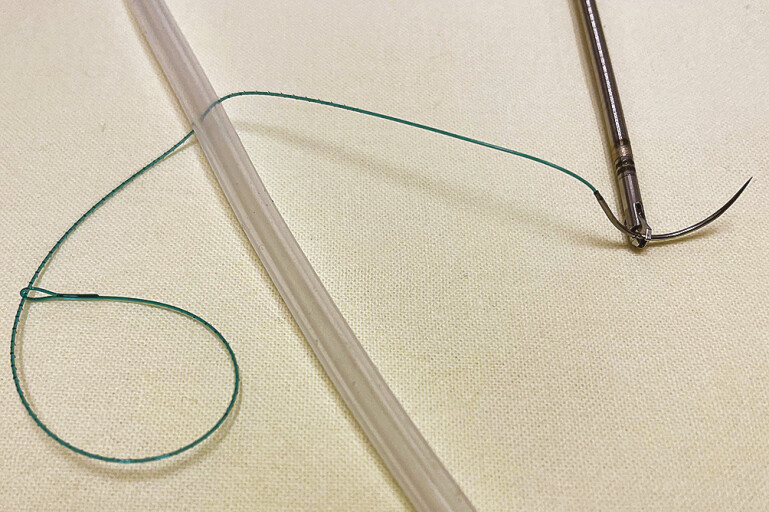
SutuArt endoscopic needle-holder grasping a needle of the V-LOC-suture material.

When suturing, the attached needle is grasped with the endoscopic needle holder at the optimal position at one-third length from the needle-basis ( Fig. 2 ). The needle can be locked in the forceps in a plane perpendicular to the scope tip. Penetration of the mucosa can be achieved by consecutive movement of the endoscope tip toward the mucosa to generate force on the needle as well as rotation of the needle-holder. Thus, the needle is forced with the adjusted timing through the tissue, until the tip of the needle appears again at the exit spot at the mucosa.

The drainage system was assembled, using a commercially available foil Suprasorb-CNP drainage film, as described by Loske 2 . All drains were small-caliber Redon-drains (4 to 6 mm).

The experiment was performed using an explanted porcine stomach with attached esophagus. Ethical aspects of the experiments were discussed. Because the explanted specimens were similar to food from a local butcher, who followed the appropriate rules in his profession, no conflicts with ethical considerations were encountered. The stomach was placed in an open box, allowing the esophagus to be stretched through a front opening and the stomach to be kept within the box during the subsequent experiments, thus enabling free maneuverability of the scope and endoscopic needle-holder within the esophagus and stomach.

**Fig. 3:**
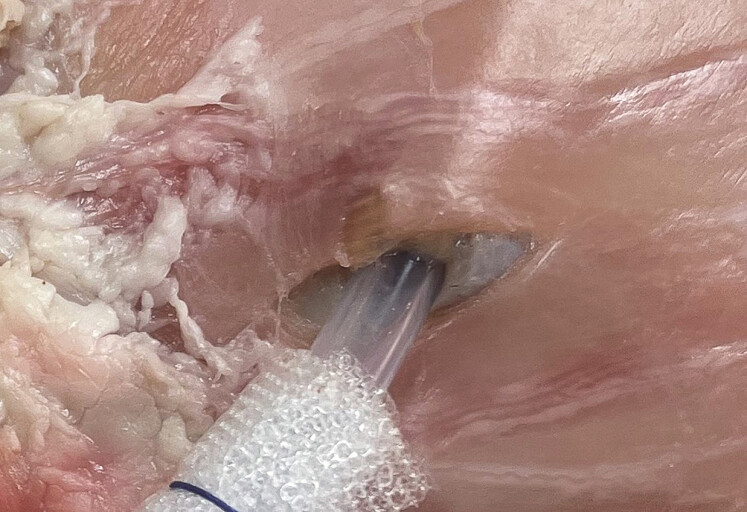
Experimental fistula site and penetration of the drain through the gastric wall; care was taken to keep this penetration site “air tight”.

A fistula position was chosen at the gastric wall for these experiments. Positions were identified at the anterior wall at the corpus and antrum and the drain was placed into the gastric wall after a minimal incision, which ensured a tight connection between the drain and the tissue, excluding any gas leak at this site ( Fig. 3 ). This was checked by introducing the scope into the gastric lumen and increasing CO _2_ -flow.

Outside the specimen, the needle holder was advanced through the working channel just outside the tip of the scope. The forceps of the needle holder was opened and the V-LOC suture was grasped with the needle with the concave side of the needle toward the scope. In this configuration, the scope with the needle-holder tip could be advanced through the esophagus into the stomach with no problems.

**Fig. 4:**
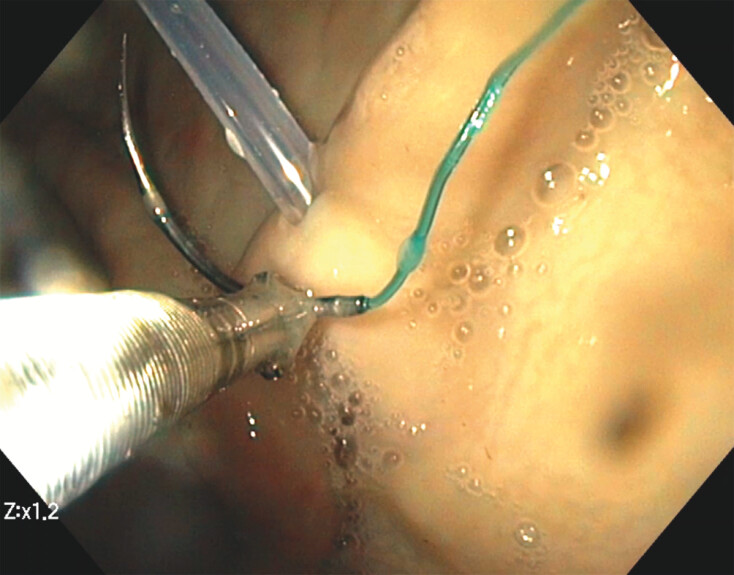
The needle of the V-LOC suture is grasped by SutuArt needle-holder at the optimal position, leaving two-thirds of the distance from the needle tip free for loading the tissue for a “big bite”.

**Fig. 5:**
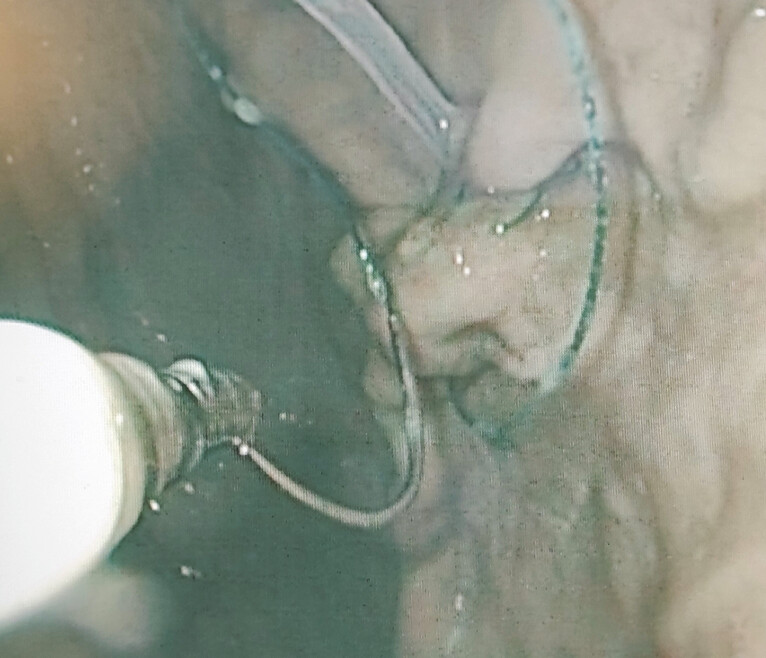
Running sutures with V-LOC to ensure safe and durable fixation of the drain at the simulated fistula site.

**Fig. 6:**
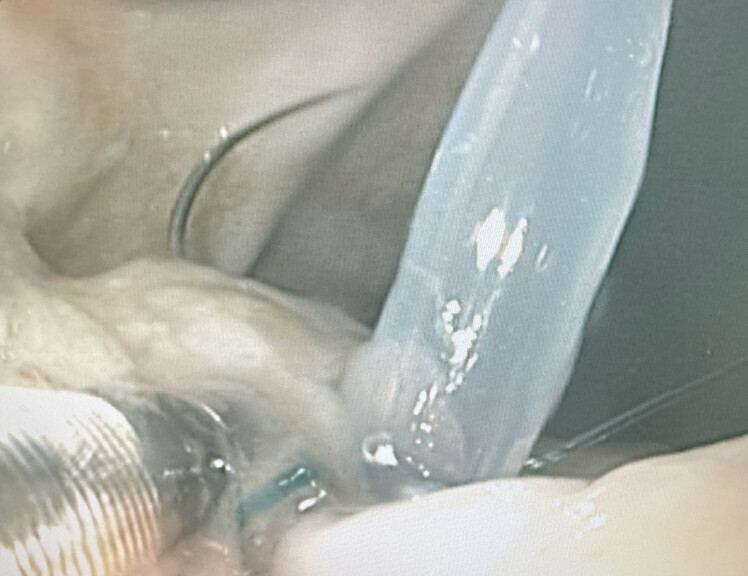
Running stitches by SutuArt needle-holder around the internal transmural drain for fixation of its position.

Inside the gastric lumen, the drain location was identified. A first stitch was placed in the close proximity to the drain to fix an initial suture position ( Fig. 4 ). Within this stitch, the attached loop of thread also was loaded and then the mucosa was penetrated, ensuring safe fixation. One stitch also had to penetrate the drain for optimal fixation. Three consecutive running stitches were performed at the drain each time to provide sufficient fixation of the drain at the “fistula” site ( Fig. 5 , Fig. 6 ).

After finishing the fixation, the V-LOC-thread was cut, leaving at least 1 cm length on both sides, one at the gastric wall and the second at the needle for better retrieval.

**Fig. 7:**
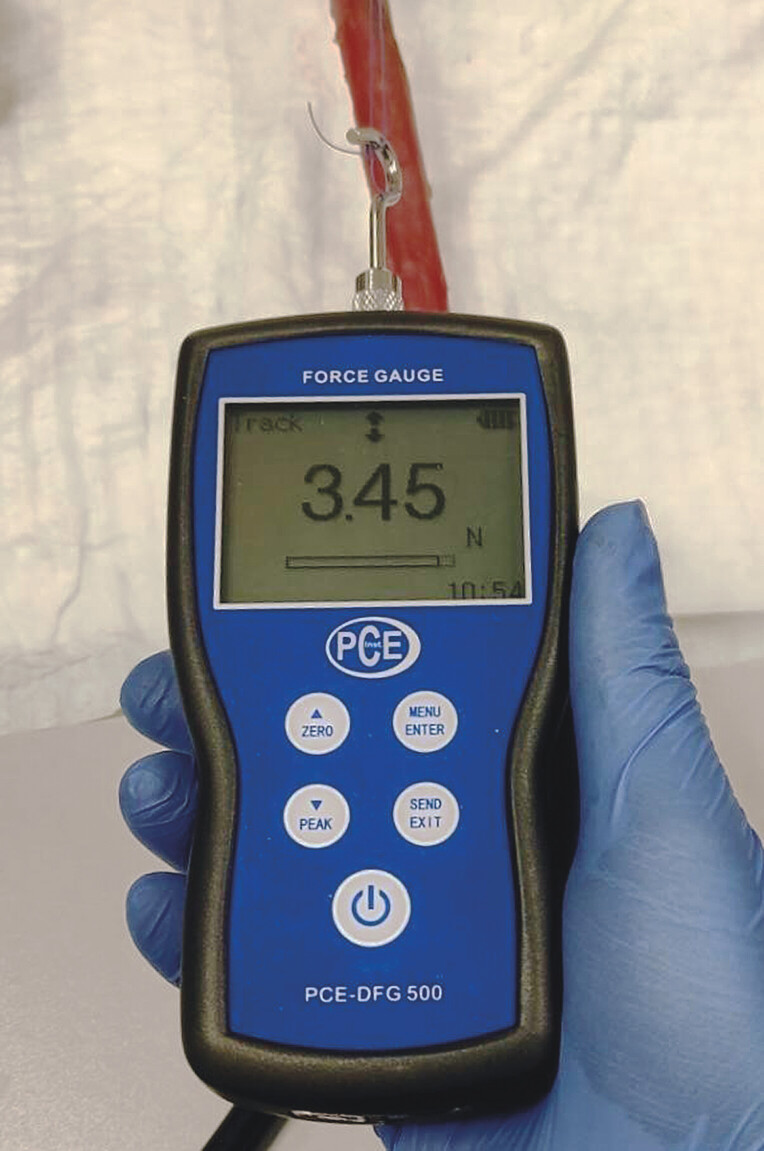
Digital force gauge continuously measuring the pulling force on the suture thread attached to the transmural drain.

Subsequently, the drain on the outside of the stomach was connected to a thread from a digital-force gauge for measuring the possible pulling force, expressed in N (Newton). The investigator pulled with increasing force until the drain started to dislocate more than 3 mm ( Fig. 7 ). At this point, a measurement was taken and documented. This force value was determined with limited dislocation. The maneuver was repeated one more time, documenting the established pulling force again. As the last test in the series, the thread was pulled with maximum force to dissolve the thread-tissue-connection and the drain was pulled out of the gastric wall, while this maximum force was also documented.

As a control test stitch, the drain was fixed with an external full-thickness hand stitch. Subsequently, the force necessary to dislocate this drain was measured.

## Results

The designed method of fixation of the drain system by using SutuArt worked in all the experiments. None of the suture fixations was insufficient in that it deteriorated from the beginning. In total, 12 tests and measurements were performed, and in all tests, three suture stitches were used in a running fashion for one fixation.

The external handmade suture fixation of the drain as three full-thickness stitches through the gastric wall (reference-stitch) showed a maximum force necessary to withstand the forced dislocation. Before complete dislocation, the plastic body of the drain was ruptured by the thread, when the pulling force reached 14.5 N. Force levels of 11.9 and 12.8 N were established during minimal dislocation (3 mm).

Subsequent test series were performed without deviation from the protocol. The procedure of fixation, starting from the first stitch to cutting the needle, had a median duration of 23 minutes (range 19–44). Table 1 shows the force values during the various tests as measured in N.

**Table:** **Table 1** Results of force-measurements by pulling on drains in their suture-fixed position in the gastric wall.

Series/test*	First pull and force at 3-mm dislocation of drain	Second pull and force at 3-mm dislocation of drain	Third pull and force at 3-mm dislocation of drain	Fourth pull and force at dissolution of fixation with complete dislocation
A	2.5	7.1	7.8	9.3
B	3.5	5.1	7.1	10.5
C	5.0	5.0	6.3	7.3
Median	3.5	5,1	7.1	9.3
*Force is expressed in newtons [n].				

Median pulling force of all tests was 6.7 N with a mean of 6.354 N. Compared with the reference value of 12 N, the running suture with 3.0V-LOC without a closing knot, just locked by the barbed-wire-structure of the thread, was able to withstand half of the reference value, generated with a full-thickness suture fixation.

When pulling force was applied until 3-mm dislocation of the drain, the median force level was 5.1 N. Median force level necessary to dissolve the suture fixation in the gastric tissue, causing complete dislocation of the drain, was 9.3 N (range 7.3–10.5 N). This value reached 64% of the maximum reference value (drain rupture).

## Discussion

This study focused on suture fixation of an internal transluminal drainage system around the clinical problem of esophageal leaks and fistulas and their management. In that regard, two major discussion points need to be addressed. The first is the technical feasibility of creating a stable position for the drain with endoscopic sutures. The second is the principle of an EID 5 8 9 . In past decades, endoscopic technology has substantially advanced the therapeutic spectrum of interventional endoscopy to help patients.

Indications for endoscopic-assisted drainage procedures as well as endoscopic-assisted stent implantations have increased 1 2 3 8 . However, risk of migration of drainage systems and stents remains an issue. Therefore, with our technical study, we explore possible uses of the new endoscopic needle holder for stable fixation of drains 5 8 9 .

Currently, there are a number of technical alternatives for suturing on the market 1 3 4 5 6 7 9 10 . Considering other suture or closure devices, it must be emphasized that the direct surgical stitch, performed by the endoscopic needle holder was favored by the authors, allowing for a stitch through the drain and gastric mucosa as a simple surgical step. Alternative techniques such as the x-tack or the Apollo-device are aiming more for an adaptation of mucosal defects 10 .

Durable positioning of the drain is most important for a patient with a fistula for two reasons. First, a stable position can alleviate the need for repetitive re-endoscopies for frequent dislocations. The second reason may be avoidance of a nasogastric tube for continuous suction This concept can only be verified by clinical studies, such as the one from Donatelli 8 .

Of course, direct closure would be an ideal option to end the problems of persisting fistulas. However, clinical experience has shown that endoscopic closure of fistulas by primary suture has not been very successful 1 9 10 . Optimal drainage is a prerequisite for long-term management of fistulas because development of granulation, stepwise healing, and durable closure of a fistula need time.

A few drawbacks of this study must be discussed. First, it is an ex-vivo experimental study to explore technical feasibility. The technical feasibility may be different in the clinical setting; however, this experimental set-up shows the mechanical strength of the SutuArt-technique with running sutures to fix a drain. The system requires a learning curve for endoscopic suturing 5 6 7 9 .

One of the shortcomings of our in-vitro experiments is lack of varying tissue conditions, which characterizes the situation in patients. The classic fistula orifice towards the gut lumen can be erosive, hard scar, or a soft inflammatory rim. In contrast to fistula closure, however, the stitch for fixation of a drain should not be placed at the soft rim but should be placed through rather normal tissue in the surroundings of the fistula to ensure safe fixation.

Furthermore, the V-LOC suture material has a resorption time span of 180 days, which has been sufficient in thousands of clinical intraluminal applications in gastrointestinal surgery and should provide stability for prolonged fistula-drainage 8 .

There are significant differences between an ex-vivo porcine stomach and a human stomach, which may limit generalizability of the findings. Despite this, there is remarkable experimental experience with endoscopic and laparoscopic training in both ex-vivo and in-vivo porcine stomachs, established in the past three decades, in which the ex-vivo settings have shown to be quite sufficient to serve as an affordable model 4 5 .

The authors decided to use the anterior wall of the stomach for placing the drain because the posterior wall of the specimen rested on the operating table. Of course, a more difficult position would have been the gastric fundus, resulting in a more demanding procedure.

Other physiologic intraluminal conditions such as peristalsis, mucus production, and possible bleeding could not be simulated and would have added to the challenge of performing these sutures, but this is rather a question of appropriate training, which has been done before with endoscopic suturing 4 9 .

The aforementioned difficulties may influence the data measured by the pulling-force gauge, and the data may not accurately represent the real stability of such suture-fixated drains in a clinical context. Nevertheless, the experiments provide a first insight into the concept of EID of chronic fistulas, stabilized by endoscopic suturing.

## Conclusions

In conclusion, this study demonstrates the conceptual possibility of using an endoscopic needle holder for suture fixation of an internal transluminal drain. Further clinical investigations are required to establish a full feasibility test of the concept.
